# 

**Published:** 1899-12

**Authors:** 


### INDEX TO VOLUME XX.

### A.

ABSTRACTS AND TRANSLATIONS:
- A Case of Triple Reimplantation, 293.
- A Simple Method of swaging Tin Plates, for Vulcanite Work, without a Swager, 97.
- Hypertrophic Gingivitis: Histological Researches, 717.
- Lithia Waters, 101.
- Naftalan, 27.
- On the Cause of So-called Phosphorous Necrosis of the Jaw in Match-Workers, 166.
- Prophylactic Inoculations, 781.
- Some Old and New Theories of Calcification, 368.
- The New Treatment of Hemorrhage, 224.
- The Relation of Dental Diseases to General Diseases, 518, 650.
- The Treatment of Suppuration by Bicarbonate of Soda, 29.

Academy of Stomatology, 114, 243, 310, 540, 742.
A Case of Triple Reimplantation, 293.
A Chinese Nail, 339.
Adaptation to Environment as shown by Cells and Tissues in Various Pathological Processes, The Power of, 359.
A Dictionary of Dental Science, 198.
Advertiser, The Ethical, 504.
A Libellous Letter, 130.
Allen, Frederick W. On Pyorrhœa Alveolaris, 438.
Alumni Association, Boston Dental College, 316.
Alumni Day at the Harvard Dental School, 691.
Alveolaris, Pyorrhœa, 438, 441, 443, 455.
Alveolaris, Pyorrhœa, Treatment of, 442, 446.
Amalgam and Oxyphosphate as Filling-Materials, 24.
A Manual of Comparative Dental Anatomy for Dental Students, 472
American Acadamy of Dental Science, 41, 171, 226, 372, 522, 605, 727.
American Dental Society of Europe, 409, 491.
American Dental Society of Japan, 617.
American Medical Association, 271.
American Medical Association, Section on Stomatology, 341.
A Method of making Seamless Gold Crowns, 162.
A Method of repairing a Broken Facing, 137.
A Method of working Cement, 72.
Amoëdo, Dr. Oscar. L'Art Dentaire en Médecine Légale, 132.
Anæsthetic, Ethyl Bromide as an, 91.
Anatomy and Histology of the Mouth and Teeth, 63.
An Epitome of the History of Medicine, 830.
Annual and Analytical Cyclopædia of Practical Medecine. By Charles E. de M. Sajous, M.D., 196, 550.
Antrum, Two Cases of Diseased, 163.
Appointments of Dentists for the Army and Navy, 72.
A Problem, Dental Education, 705.
A Process for Nickel Plating, 137.
A Proper Food Standard in its Relation to the Teeth, 582.
A Reply to Dr. Talbot, 134.
A Review of Recent Legal Discussions affecting Physicians, Dentists, Druggists, etc. By W. A. Purrington, 689.
Are We punishing the Right Man? 511.
Army and Navy, Appointments of Dentists for, 72.
Army and Navy, Dental Surgeons in the, 773.
Army and Navy Dental Surgery, 716.
Army and Navy, Dentists in the, 192.
Army Dentists, 355.
Arnone, Dr. Luigi. On Hypertrophic Gingivitis: Histological Researches, 717.
Arsenic in devitalizing Pulps, 406.
Artificial Nose of Celluloid, 211.
Artistic Dentistry, 71.
A Simple Method of swaging Tin Plates, for Vulcanite Work, without a Swager, 97.
A Substitute for Rubber Gloves, 72.

### B.

Baker, Dr. George T. On Dental Surgeons in the Army and Navy, 773.
- On Formaldehyde in Dentistry, 221.

Barrett, A Letter from Dr., 268.

Bennett, Mr. J. F. On Some Old and New Theories of Calcification, 368.
BIBLIOGRAPHY:
A Dictionary of Dental Science and such Words and Phrases of the Collateral Sciences as pertains to the Art and Practice of Dentistry. By Chapin A. Harris, M.D., D.D.S., 198.
A Manual of Comparative Dental Anatomy for Dental Students. By Alton Howard Thompson, D.D.S., 472.
Anatomy and Histology of the Mouth and Teeth By I. Norman Broomell, D.D.S., 63.
An Epitome of the History of Medicine, 830.
Annual and Analytical Cyclopædia of Practical Medicine. By Charles E. de M. Sajous, M.D., 196, 550.
A Review of Recent Legal Decisions, affecting Physicians, Dentists, Druggists, etc. By W. A. Purrington, 689.
Chemistry and Metallurgy applied to Dentistry. By Vernon J. Hall, Ph.D., 690.
Degeneracy: Its Causes, Signs, and Results. By Eugene S. Talbot, M.D., D.D.S., 330.
Interstitial Gingivitis; or, So-called Pyorrhœa Alveolaris. By Eugene S. Talbot, M.D., D.D.S., 685.
L'Art Dentaire en Médecine Légale. By Dr. Oscar Amoëdo, Paris, 132.
Lehrbuch der Conservirenden Zahnheilkunde. Von W. D. Miller, 66.
Methods of filling Teeth. By R. Ottolengui, M.D.S., 333.
The Hygiene of the Mouth. By R. Denison Pedley, F.R.C.S., L.D.S., 831.
Three Thousand Questions on Medical Subjects arranged for Self-Examination, 267.
Transactions of the Iowa State Dental Society at the Thirty-fifth Annual Meeting, held at Des Moines, May 3, 4, 5, and 6, 1898, 268.
Bicarbonate of Soda, Treatment of Suppuration by, 29.
Bishop, Burton F. On Pyorrhœa Alveolaris, 443.
Bleeding Gums, 406.
Boenning, Henry C., M.D. On Clinical Studies of Some Suppurative Diseases of the Maxillæ, 146.
Bonwill, Death of Dr. W. G. A., 684.
Bonwill, Dr. W. G. A., 764.
Bonwill, Reminiscences of Dr., 714.
Borax, Solution for Soldering, 137.
Boston Dental College, 618.
Broken Facings, A Method of repairing, 137.
Bromide, Ethyl, as an Anæsthetic, 91.
Brooks, George, D.D.S Three Years none too long for a Dental Course, 778.
Broomell, I. Norman, D.D.S. On Anatomy and Histology of the Mouth and Teeth, 63.
Brubaker, Dr. Albert P. On the Neuron Conception as a Means of interpreting Reflex Disorders due to Dental Irritation, 493.
Bryan, Dr. L. C. On securing Loose Teeth affected with Pyorrhœa, 436.
Bur-Excavation, 136.

C.

Calcification, Old and New Theories of, 368.
California State Board of Dental Examiners, 489.
Carbolic Acid Poisoning, Treatment of, 620.
Carbonized Cotton as a Root-Canal Filling, 695.
Cardwell, Harold. On Pyorrhœa Alveolaris, 445.
Case of Triple Reimplantation, 293.
Cataphoresis—Leakage, 406.
Catching, B. H., D.D.S. Army and Navy Dental Surgery, 716.
Celluloid, Artificial Nose of, 211.
Cement, A Method of working, 72.
Cements, Mechanical Tests applied to Dental, 699.
Central Dental Association of Northern New Jersey, 492.
Changes, Journalistic, 274.
Chemistry and Metallurgy applied to Dentistry, 690.
Chicago Dental Society, 411.
Chinese Nail, A, 339
Circular Letter of the Foreign Relations Committee of the National Association of Dental Faculties of America, 482.
Clacking of Full Dentures, 211.
Clark, R. H., D.D.S. On Turpentine, 817.
Clinical Studies of some Suppurative Diseases of the Maxillæ, 146.
Cocaine and Eucaine, 834.
Cocaine, Unusual Effect of, 771.
Color in Implantation, 340.
Colorado State Dental Association, 624.
Comparative Method of teaching Dental Anatomy, 30.
Conservatism, Therapeutic, 619.
Conventions, 466.
Correction, 618, 685, 772, 830.
Cotton, Carbonized, as a Root Canal Filling, 695.
Criticism, Western, 327.
Criticism, What constitutes Just? 264.
Crown, The Meriam Extension, 141.
Crowns, Method of making Seamless Gold, 162.
Crowns, Repairing Gold, 340.

CURRENT NEWS:
American Dental Society of Europe, 409, 491.
American Medical Association, Section on Stomatology, 341.
California State Board of Dental Examiners, 489.
Central Dental Association of Northern New Jersey, 492.
Chicago Dental Society, 411.
Circular Letter of the Foreign Relations Committee of the National Association of Dental Faculties of America, 482.
Colorado State Dental Association, 624.
Correction, 772.
Dental Association of New South Wales, 343.
Dental Commissioners of Connecticut —Legal Notice, 343.
Dental Society, State of New York, 276, 491.
Harvard Dental Alumni Association, 556.
International Dental Congress, 837.
Maine Dental Society, 491.
Maryland State Dental Association, 838.
Missouri State Dental Association, 411, 838.
National Association of Dental Examiners, 488.
National Association of Dental Fulculties and the National Association of Dental Examiners, 621.
National Dental Association, 407, 490, 623.
National School of Dental Technics, 838.
New England Association of Dental Examiners, 275.
New Jersey Dental Examining Board, 412.
New Jersey State Dental Society, 489.
Odontological Society of Rockford, Ill., 139.
Ohio State Dental Society, 140, 837.
Oregon State Dental Association, 212.
Pennsylvania Board of Dental Examiners, 772.
South Dakota State Dental Society, 344, 624.
Southern Branch of the National Dental Association, 138.
Tennessee Dental Association, 492.
The National Dental Association—Committee on History, 342.
To the Alumni of the Dental Department of the University of Maryland, 555.
Union Meeting, Seventh and Eighth District Dental Societies, State of New York, 696.
Wisconsin State Dental Society, 489.
Cutting Wisdom-Tooth, Death from, 771.

D.

Dangers in Fruit, Some of the, 71.
Darby, Edwin T, D.D.S. On Dental Education a Problem, 705.
On Porcelain Inlays as made by Dr. Jenkins, of Dresden, 73.
Dawbarn, Robert H. M., M.D. On False Tonsils, 345.
Death from Attempt at Tooth-Extraction, 481.
Death from cutting Wisdom-Tooth, 771.
Death from Hemorrhage caused by lancing Gums, 769.
Death of Dr. B. H. Catching, 829.
Death of Dr. W. G. A. Bonwill, 684.
Decision upon Jury Services, 471.
Degeneracy: Its Causes, Signs, and Results, 330.
Delabarre, Frank A., M.D., D.D.S. On The Power of Adaptation to Environment as shown by Cells and Tissues in Various Pathological Processes, 359.
Delicacy in Operating, 736.
Dental Anatomy, The Comparative Method of teaching, 30.
Dental Association of New South Wales, 343.
Dental Cements, Mechanical Tests applied to, 699.
Dental Colleges, The *Dental Digest* and, 469.
Dental Commissioners of Connecticut—Legal Notice, 343.
*Dental Digest* and Dental Colleges, The, 469.
Dental Diseases, The Relations of, to General Diseases, 518.
Dental Disorders, The Predisposing Factor in, 277.
Dental Education a Problem, 705.
Dental Examinations in the United States, Some Suggestions in regard to, 362.
Dental Faculties, The National Association of, 472.
Dental Irritation, The Neuron Conception as a Means of interpreting Reflex Disorders due to, 493.
Dental Journals, Suspension of, 62.
Dental Knowledge in the Medical Profession, 479.
Dental Legislation, 571.
Dental Lesions and their Relation to Nasal and Accessory Cavities, 625.
Dental Notes, 433, 648, 697, 777.
Dental Organizations in the Untted States: Their Mistakes and Failures, 557.
Dental Practice, The Relation of Science to Progress in, 78.
Dental Protective Association, The, 679.
Dental Science in 1480, as expounded by Peter De Largelata, 712.
Dental Society, State of New York, 276, 491.
Dental Surgeons in the Army and Navy, 773.

Dental Surgery, Army and Navy, 716.
Dentine, Klein's Plastic, 770.
Dentine, The Non-Removal of Softened, 210.
Dentistry, Artistic, 71.
Dentistry, Formaldehyde in, 221.
Dentistry, Honorary Degrees in,—Why Not? 477.
Dentistry in State Institutions, 763.
Dentistry, Prophylaxis in, 16.
Dentistry, The History of, 330.
Dentistry, the Value of the Doctorate in, 400.
Dentists, Army, 355.
Dentists for the Army and Navy, Appointments of, 72.
Dentists in the Army and Navy, 192.
Dentures, Clacking of Full, 211.
Deposits, Tincture of Iodine for Removal of, 406.
Destruction by Fire, 822.
Devitalizing Pulps, Arsenic in, 406.
Dickinson, E. B., D.D.S. On Ethyl Bromide as an Anæsthetic, 91.
Discolored Teeth, The Question of Priority in the Introduction of the Process of Bleaching, 475.
Diseased Antrum, Two Cases of, 163.
Diseases of the Maxillæ, Clinical Studies of some Suppurative, 146.
Diseases of the Teeth, Systemic Remedies in, 289.
Display of Gold in Teeth, 340.
Doctorate in Dentistry, The Value of the, 400.
Does License to practise Medicine permit the Practice of Oral Surgery? 478
Domestic Correspondence:
- Alumni Day at the Harvard Dental School, 691.
- American Medical Association, 271.
- A Reply to Dr. Talbot, 134.
- Criticism upon the Use of "Volasen," 833.
- Death from Hemorrhage caused by lancing Gums, 769.
- Double Resection of the Lower Maxilla for Protruding Lower Jaw, 337.
- Dr. Angle's Reply to Dr. Talbot, 551.
- Honorary Degrees in Dentistry,—Why Not? 477.
- Klein's Plastic Dentine, 770.
- Letter from Dr. Barrett, 268.
- National or International? 69.
- Reply to Editorial in February Number, 270.
- Report of the Foreign Relations Committee, 200.
- The Medico-Chi wins, 339.
- Work of the Foreign Relations Committee, 403.

Double Resection of the Lower Maxilla for Protruding Lower Jaw, 337.
Do we wish to be Medical Specialists? 480.
Dr. Angle's Reply to Dr. Talbot, 551.
Dr. Bonwill, Reminiscences of, 714.
Durability of Cement Fillings, 835.
Duty, Indifference to Professional, 59.

E.

Editorial:
- A Libellous Letter, 130.
- Correction, 618, 685, 830.
- Death of Dr. B. H. Catching, 829.
- Death of Dr. W. G. A. Bonwill, 684.
- Decision upon Jury Services, 471.
- Dentistry in State Institutions, 763.
- Dentists in the Army and Navy, 192.
- Destruction by Fire, 822.
- Entrance Qualifications, 759.
- Example is the School of Mankind, 547.
- Horace Wells, 829.
- Indifference to Professional Duty, 59.
- International Dental Congress, Paris, 1900, 63.
- Is Dentistry a Remunerative Profession? 324.
- National Association of Dental Faculties, 616.
- Niagara Falls, 1899, 614.
- Practical Results of Higher Education, 126.
- Retrospection, 823.
- Special Notice, 471.
- Suspension of Dental Journals, 62.
- The American Dental Society of Japan, 617.
- The Boston Dental College, 618.
- The Conventions, 466.
- The *Dental Digest* and Dental Colleges, 469.
- The Dental Protective Association, 679.
- The Foreign Relations Committee, 194, 825.
- The History of Dentistry, 330.
- The National Association of Dental Faculties, 472.
- The Refusal of Wisconsin, 682.
- The Value of the Doctorate in Dentistry, 400.
- Western Criticism, 327.
- What constitutes Just Criticism? 264.

Education a Problem, Dental, 705.
Education, Practical Results of Higher, 126.
Effect of Cocaine, Unusual, 771.
Egotist, The, 275.
Engs, John S., D.D.S. On Pains from Malocclusion, 779.
Entrance Qualifications, 759.
Ethical Advertiser, The, 504.
Ethyl Bromide as an Anæsthetic, 91.
Example is the School of Mankind, 547.
Excavation, Bur-, 136.
Extension Crown, The Meriam, 141.
Extraction, Death from Attempt at Tooth-, 481.
Extraordinary Results, 136.

F.

Facings, A Method of repairing Broken, 137.
Facts *versus* Teaching, 286.
Faculties, The National Association of Dental, 472.
Fads and Faddists, 835.
False Tonsils, 345.
Filling, Carbonized Cotton as a Root-Canal, 695.
Filling-Materials, Amalgam and Oxyphosphate as, 24.
Fire, Destruction by, 822.
Flanagan, A. J., D.D.S. On Infectious Dentistry, 813.
Food Standard, A Proper, 582.
Foreign Correspondence;
 Letter from Dr. N. S. Jenkins, Dresden, 68.
 The Question of Priority in the Introduction of the Process of bleaching Discolored Teeth, 475.
Foreign Relations Committee, Report of the, 200.
Foreign Relations Committee, The, 194, 826.
Foreign Relations Committee, Work of the, 403.
Formaldehyde in Dentistry, 221.
Full Dentures, Clacking of, 211.

G.

Geran, J. P., D.D.S., 474.
Glue, Liquid, 212.
Gold Crowns, A Method of making Seamless, 162.
Gold Crowns, Repairing, 340.
Gold in Teeth, The Display of, 340.
Gout, 424.
Gray, George R., D.D.S., D.M.D. On A Proper Food Standard, 582.
Greenbaum, Leo, M.D., D.D.S. On Systemic Remedies in Diseases of the Teeth, 289.
Gums, Bleeding, 406.

H.

Hall, Vernon J., Ph.D. Chemistry and Metallurgy applied to Dentistry, 690.
Harris, Chapin A., M.D., D.D.S. A Dictionary of Dental Science, etc., 198.
Harvard Dental Alumni Association, 556.
Harvard Dental School, Alumni Day at the, 691.
Hayter, Dr. Mark. On A Method of making Seamless Gold Crowns, 162.
Hemorrhage caused by lancing Gums, Death from, 769.
Hemorrhage, The New Treatment for, 224.
Heyke, John E. On Treatment of Pyorrhœa Alveolaris, 442.
Higher Education, Practical Results of, 126.
Hinderances, Vocal, 213.
Histology of the Mouth and Teeth, Anatomy and, 63.
History of Dentistry, 330.
Hodgkin, Dr. J. B. On Are we punishing the Right Man? 511.
Honorary Degrees in Dentistry,—Why Not? 477.
Horace Wells, 829.
Hubbard, Dwight L., M.D. On Vocal Hinderances, 213.
Hunter, William, M.D. On The Relations of Dental Diseases to General Diseases, 518, 650.
Hypertrophic Gingivitis: Histological Researches, 717.

I.

Implantation, Color in, 340.
Impressions, Use of Tin-Foil in Model and Bite, 211.
Independent Journalism, 478.
Indifference to Professional Duty, 59.
Infectious Dentistry, 813.
Information, 274.
Inlays, Porcelain, as made by Dr. Jenkins, of Dresden, 73.
Institutions, Dentistry in State, 763.
International Dental Congress, Paris, 1900, 63, 837.
International, National or? 69.
Interstitial Gingivitis; or So-called Pyorrhœa Alveolaris, 685.
Iodine and Nitrate of Silver Stains, Removal of, 620.
Iodine for Removal of Deposits, 406.
Is Dentistry a Remunerative Profession? 324.
Is the Enamel a Vital Tissue? 621.

J.

Jameson, G. L. S., D.D.S. On Dental Lesions and their Relation to Nasal and Accessory Cavities, 625.
Jenkins, Dr. N. S., Dresden, Letter from, 68.
Jenkins, Porcelain Inlays as made by Dr., of Dresden, 73.
Journalism, Independent, 478.
Journalistic Changes, 274.
Journals, Suspension of Dental, 62.
Jury Services, Decision upon, 471.

K.

Keith, Henry Howard, D.D.S., 335.
Kirk, Edward C., D.D.S. On the Predisposing Factor in Dental Disorders, 277.
Klein's Plastic Dentine, 770.

Knowles, Vernon, L.D.S. On A Simple Method of swaging Tin Plates, for Vulcanite Work, without a Swager, 97.

L.

Lactic Acid Injection, Necrosis from, 220.
L'Art Dentaire en Médecine Légale, 132.
Lead-Tin and Lead-Antimony Alloys, Quick Testing, 480.
Leakage, Cataphoresis, 406.
Legislation, Dental, 571.
Lehrbuch der Conservirenden Zahnheilkunde. By Von W. D. Miller, 66.
Lesions, Dental, and their Relation to Nasal and Accessory Cavities, 625.
Lesions of Syphilis of the Mouth, 576.
Letter, A Libellous, 130.
Letter from Dr. Barrett, 268.
Letter from Dr. N. S. Jenkins, Dresden, 68.
Libellous Letter, A, 130.
Liquid Glue, 212.
Lithia Waters, 101.
Loretin, 836.
Lower Molars, Reflexes from, 641.

M.

Maine Dental Society, 491.
Malocclusion, Pains from, 779.
Maryland State Dental Association, 838.
Massachusetts Dental Society, 752.
McCutcheon, W. H., D.D.S. On Two Cases of Diseased Antrum, 163.
Maxillæ, Clinical Studies of some Suppurative Diseases of the, 146.
Mechanical Tests applied to Dental Cements, 699.
Medical Specialists, Do we wish to be? 480.
Medicine, An Epitome of the History of, 830.
Medico-Chi wins, The, 339.
Meriam, Dr. Horatio C. On The Meriam Extension Crown, 141.
Meriam Extension Crown, The, 141.
Method of making Seamless Gold Crowns, 162.
Method of repairing a Broken Facing, 137.
Method of working Cement, A, 72.
Methods of filling Teeth, 333.
Miller, Von, W. D. Lehrbuch der Conservirenden Zahnheilkunde, 66.
Missouri State Dental Association, 411, 838.
Molars, Reflexes from Lower, 641.
Mouldine, 619.
Mouth, Lesions of Syphilis of, 576.
Mouth and Teeth, Anatomy and Histology of the, 63.

N.

Naftalan, 27.
Nail, A Chinese, 339.
National Association of Dental Examiners 488.
National Association of Dental Faculties, 472, 616, 666.
National Association of Dental Faculties and National Association of Dental Examiners, 621.
National Dental Association, 30, 138, 407, 490, 611, 623, 657, 722, 788.
National Dental Association, Committee, on History, 342.
National or International? 69.
National School of Dental Technics, 838.
Navy, Appointments of Dentists for Army and, 72.
Navy, Dentists in the Army and, 192.
Necrosis from Lactic Acid Injection, 220.
Necrosis of the Jaw in Match-Workers, On The Cause of So-called, 166.
Neuron Conception as a Means of interpreting Reflex Disorders due to Dental Irritation, The, 493.
New England Association of Dental Examiners, 275.
New Jersey Dental Examining Board, 412.
New Jersey State Dental Society, 489.
New Treatment for Hemorrhage, 224.
New York Institute of Stomatology, The, 102, 140, 175, 235, 295, 385, 449, 585, 796.
Niagara Falls, 1899, 614.
Nickel Plating, A Process for, 137.
Non-Removal of Softened Dentine before filling, 210.
Northeastern Dental Association, 51, 118, 253, 696.
Nose of Celluloid, Artificial, 211.
NOTES AND COMMENTS:
A Chinese Nail, 339.
A Method of repairing a Broken Facing, 137.
A Method of working Cement, 72.
Appointments of Dentists for the Army and Navy, 72.
A process for Nickel Plating, 137.
A substitute for Rubber Gloves, 72.
Arsenic in devitalizing Pulps, 406.
Artificial Nose of Celuloid, 211.
Artistic Dentistry, 71.
Bleeding Gums, 406.
Borax Solution for Soldering, 137.
Bur-Excavation, 136.
Carbonized Cotton as a Root-Canal Filling, 695.
Cataphoresis and Arsenic, 837.
Cataphoresis—Leakage, 406.
Clacking of Full Dentures, 211.
Cocaine and Eucaine, 834.
Color in Implantation, 340.
Death from Attempt at Tooth-Extraction, 481.
Death from cutting Wisdom-Tooth, 771.
Delicacy in Operating, 136.
Dental Knowledge in the Medical Profession, 479.

Notes and Comments:
Does License to practice Medicine permit the Practice of Oral Surgery? 478.
Do we wish to be Medical Specialists? 480.
Durability of Cement Fillings, 835.
Extraordinary Results, 136.
Fads and Faddists, 835.
Independent Journalism, 478.
Information, 274.
Is the Enamel a Vital Tissue? 621.
Journalistic Changes, 274.
Lead-Tin and Lead-Antimony Alloys, Quick Testing, 480.
Liquid Glue, 212.
Loretin, 836.
Novel Method of filling Teeth, 694.
Office of the State Societies, 694.
Professional Recruits, 275.
Removal of Iodine and Nitrate of Silver Stains, 620.
Repairing Gold Crowns, 340.
Some of the Dangers in Fruit, 71.
The Display of Gold in Teeth, 340.
The Egotist, 275.
The Non-Removal of Softened Dentine before filling, 210.
Therapeutic Conservatism, 619.
Time Limit in Professional Work, 620.
Tincture of Iodine for the Removal of Deposits, 406.
Treatment of Carbolic Acid Poisoning, 620.
Unusual Effect of Cocaine, 771.
Use of Tin-Foil in Model and Bite Impressions, 211.
Notes, Dental, 433, 648, 697, 777.
Notice, Special, 471.
Novel Method of filling Teeth, 694.

O.

Obituary:
Bonwill, Dr. W. G. A, 684, 714, 764.
Geran, J. P., D.D.S., 474.
Keith, Henry Howard, D.D.S., 335.
Southwick, A. P., D.D.S., 475.
Odontological Society of Rockford, Ill., 139.
Office of the State Societies, 694.
Ohio State Dental Society, 140, 837.
Old and New Theories of Calcification, 368.
On the Cause of So-called Phophorous Necrosis of the Jaw in Match-Workers, 166.
Operating, Delicacy in, 136.
Oregon State Dental Association, 212.
Original Communications:
Amalgam and Oxyphosphates as Filling-Materials, 24.
A Method of making Seamless Gold Crowns, 162.

Original Communications:
A Proper Food Standard, 582.
Are we punishing the Right Man? 511.
Army and Navy Dental Surgery, 716.
Army Dentists, 355.
Clinical Studies of some Suppurative Diseases of the Maxillæ, 146.
Dental Education a Problem, 705.
Dental Legislation, 571.
Dental Lesions and their Relation to Nasal and Accessory Cavities, 625.
Dental Notes, 433, 648, 697, 777.
Dental Organizations: Their Mistakes and Failures, 517.
Dental Science in 1480, as expounded by Peter de Largelata, 712.
Dental Surgeons in the Army and Navy, 773.
Ethyl Bromide as an Anæsthetic, 91.
Facts *versus* Teaching, 286.
False Tonsils, 345.
Formaldehyde in Dentistry, 221.
Gout, 424.
Lesions of Syphilis of the Mouth, 576.
Mechanical Tests applied to Dental Cements, 699.
Necrosis from Lactic Acid Injection, 220.
Pains from Malocclusion, 779.
Porcelain Inlays as made by Dr. Jenkins, of Dresden, 73.
Prophylaxis in Dentistry, 16
Pyorrhœa Alveolaris, 438, 441, 443, 445.
Pyorrhœa Alveolaris, from a Bacteriological Stand-Point, with a Report of some Investigations, and Remarks on the Treatment, 413.
Reflexes from Lower Molars, 641.
Remarks—Truman, 447.
Reminiscences of Dr. Bonwill, 714.
Root-Treatment: A Positive Method, 152.
Securing Loose Teeth affected with Pyorrhœa, 436.
Some Suggestious in regard to Dental Examinations in the United States, 362.
Systemic Remedies in Diseases of the Teeth, 289.
The Ethical Advertiser, 504.
The Meriam Extension Crown, 141.
The Neuron Conception as a Means of interpreting Reflex Disorders due to Dental Irritation, 493.
The Power of Adaptation to Environment as shown by Cells and Tissues in Various Pathological Processes, 359.
The Predisposing Factor in Dental Disorders, 277.
The Relation of Science to Progress in Dental Practice, 78.
Three Years none too long for a Dental Course, 778.

ORIGINAL COMMUNICATIONS:
"Tothe-Lore," 1.
Treatment of Pyorrhœa Alveolaris, 442.
Two Cases of Diseased Antrum, 163.
Vocal Hinderances, 213.
Ottolengui, R., M.D.S. On Methods of filling Teeth, 333.
Oxyphosphate and Amalgam as Filling-Materials, 24.

P.

Pains from Malocclusion, 779.
Palmer, Dr. S. B. On Facts *versus* Teaching, 286.
On The Relation of Science to Progress in Dental Practice, 78.
Park, Roswell, A.M., M.D. An Epitome of the History of Medicine, 830.
Parmele, George L., M.D., D.M.D. On "Tothe-Lore," 1.
Pedley, R. Denison, F.R.C.S., L.D.S. The Hygiene of the Mouth, 831.
Pennsylvania Board of Dental Examiners, 772.
Phosphorous Necrosis of the Jaw in Match-Workers, On the Cause of So-called, 166.
Plastic Dentine, Klein's, 770.
Plating, A Process for Nickel, 137.
Pond, Virgil C, B.P., D.M.D. On Mechanical Tests applied to Dental Cements, 699.
Porcelain Inlays as made by Dr. Jenkins, of Dresden, 73.
Positive Method, Root-Treatment: A, 132.
Practical Results of Higher Education, 126
Predisposing Factor in Dental Disorders, The, 277.
Process for Nickel Plating, 137.
Professional Duty, Indifference to, 59.
Professional Recruits, 275.
Professional Work, Time Limit in, 620.
Progress in Dental Practice, Relation of Science to, 78.
Proper Food Standard, A, 582.
Prophylactic Inoculations, 781.
Prophylaxis in Dentistry, 16.
Protective Association, The Dental, 679.
Protruding Lower Jaw, Double Resection of the Lower Maxilla for, 337.
Pulps, Arsenic in devitalizing, 406.
Punishing the Right Man, Are we? 511.
Purrington, W. A. A Review of Recent Legal Decisions affecting Physicians, Dentists, Druggists, etc, 689.
Pyorrhœa Alveolaris, 438, 441, 443, 445.
Pyorrhœa Alveolaris, from a Bacteriological Stand-Point, with a Report of some Investigations, and Remarks on the Treatment, 413.
Pyorrhœa Alveolaris, Treatment of, 442.
Pyorrhœa, Securing Loose Teeth affected with, 436.
Pyorrhœa, Treatment in Detail of, 446.

Q.

Qualifications, Entrance, 759.

R.

Recruits, Professional, 275.
Reflex Disorders due to Dental Irritation, The Neuron Conception as a Means of interpreting, 493.
Reflexes from Lower Molars, 641.
Refusal of Wisconsin, The, 682.
Reimplantation, A Case of Triple, 293.
Relation of Science to Progress in Dental Practice, 78.
Remarks, 447.
Remarks on the Treatment of Pyorrhœa Alveolaris, 413.
Remedies in Diseases of the Teeth, Systemic, 289.
Reminiscences of Dr. Bonwill, 714.
Remunerative Profession, Is Dentistry a? 324.
Removal of Deposits, Tincture of Iodine for, 406.
Removal of Iodine and Nitrate of Silver Stains, 620.
Repairing Broken Facings, A Method of, 137.
Repairing Gold Crowns, 340.
Reply to Dr. Talbot, A, 134.
Reply to Editorial in February Number, 270.
Report of the Foreign Relations Committee, 200.
REPORTS OF SOCIETY MEETINGS:
Academy of Stomatology, 114, 243, 310, 540, 742.
Alumni Association, Boston Dental College, 316.
American Academy of Dental Science, 41, 171, 226, 372, 522, 605, 727.
Massachusetts Dental Society, 752, 813.
National Association of Dental Faculties, 666.
National Dental Association, 30, 611, 657, 722, 788.
New York Institute of Stomatology, The, 102, 140, 175, 235, 295, 385, 449, 585, 796.
Northeastern Dental Association, 51, 118, 253, 696.
Results, Extraordinary, 136.
Results of Higher Education, Practical, 126.
Retrospection, 823.
Rogers, F. T., M.D. On the Ethical Advertiser, 504.
Rollins, William. On Dental Notes, 433, 648, 697, 777.
Root-Canal Filling, Carbonized Cotton as a, 695.
Root-Treatment: A Positive Method, 152.
Rosenbaum, Dr. On Naftalan, 27.

Royce, Waldo E., D.D.S. On Some Suggestions in regard to Dental Examinations in the United States, 362.
Rubber Gloves, A Substitute for, 72.
Rubber, Some Properties of Vulcanite, 579.

S.

Sajous, Charles E de M., M.D. Annual and Analytical Cyclopædia of Practical Medicine, 196, 550.
Schamberg, Jay F., A.B., M.D. On Gout, 424.
Schamberg, Morris, I., D.D.S., M.D. On Army Dentists, 355.
School of Mankind, Example is the, 547.
Seamless Gold Crowns, Method of making, 162.
Securing Loose Teeth affected with Pyorrhœa, 436.
Simple Method of swaging Tin Plates, A, for Vulcanite Work, without a Swager, 97.
Smith, D. D., D.D.S., M.D. On Prophylaxis in Dentistry, 16.
Smith, F. Milton, D.D.S. On Root-Treatment: A Positive Method, 152.
Smith, Theo. V. On Pyorrhœa Alveolaris, 443.
Societies, Office of the State, 694.
Soda, Bicarbonate of, Treatment of Suppuration by, 29.
Soldering, A Borax Solution for, 137.
Solution for Soldering, A Borax, 137.
Some of the Dangers in Fruit, 71.
Some Old and New Theories of Calcification, 368.
Some Suggestions in regard to Dental Examinations in the United States, 362.
South Dakota State Dental Society, 344.
Southern Branch of the National Dental Association, 138.
Southern Dental Association, 623.
Southwick, A. P., D.D.S., 475.
Southwick, George R., M.D. On Lesions of Syphilis of the Mouth, 576.
Special Notice, 471.
State Societies, Office of the, 694.
Stevenson, Frederick A., D.M.D. On Dental Legislation, 571.
Stockman, Ralph, M.D. On the Cause of So-called Phosphorous Necrosis of the Jaw in Match-Workers, 166.
Strang, Dr. C. W. On Amalgam and Oxyphosphates as Filling-Material, 24.
Substitute for Rubber Gloves, 72.
Suppuration, The Treatment of, by Bicarbonate of Soda, 29.
Suppurative Diseases of the Maxillæ, Clinical Studies of some, 146.
Surgery, Army and Navy Dental, 716.
Suspension of Dental Journals, 62.
Swaging Tin Plates, for Vulcanite Work, without a Swager, A Simple Method for, 97.
Syphilis of the Mouth, Lesions of, 576.
Systemic Remedies in Diseases of the Teeth, 289.

T.

Talbot, Dr., A Reply to, 134.
Talbot, Eugene S., M.D., D.D.S. On Degeneracy: Its Causes, Signs, and Results, 330.
  Interstitial Gingivitis; or, So-called Pyorrhœa Alveolaris, 685.
  On Reminiscences of Dr. Bonwill, 714.
Teaching, Facts *versus*, 286.
Teeth affected with Pyorrhœa, Securing, 436.
Teeth, Anatomy and Histology of the Mouth and, 63.
Teeth, Novel Method of filling, 694.
Teeth, Systemic Remedies in Diseases of the, 289.
Teeth, The Display of Gold in, 340.
Teeth, The Question of Priority in the Introduction of the Process of bleaching Discolored, 475.
Tennessee Dental Association, 492.
Tests, Mechanical, applied to Dental Cements, 699.
The Comparative Method of teaching Dental Anatomy, 30.
The Conventions, 466.
The *Dental Digest* and Dental Colleges, 469.
The Dental Protective Association, 679.
The Display of Gold in Teeth, 340.
The Egotist, 275.
The Ethical Advertiser, 504.
The Foreign Relations Committee, 194, 825.
The History of Dentistry, 330.
The Meriam Extension Crown, 141.
The Medico-Chi wins, 339.
The National Dental Association—Committee on History, 342.
The National Association of Dental Faculties, 472.
The Neuron Conception as a Means of interpreting Reflex Disorders due to Dental Irritation, 493.
The New Treatment of Hemorrhage, 224.
The Non-Removal of Softened Dentine before filling, 210.
Theories of Calcification, Some Old and New, 368.
The Power of Adaptation to Environment as shown by Cells and Tissues in Various Pathological Processes, 359.
The Predisposing Factor in Dental Disorders, 277.
The Question of Priority in the Introduction of the Process of bleaching Discolored Teeth, 475.
Therapeutic Conservatism, 619.
The Refusal of Wisconsin, 682.

The Relation of Science to Progress in Dental Practice, 78.
The Relations of Dental Diseases to General Diseases, 518, 650.
The Treatment of Suppuration by Bicarbonate of Soda, 29.
The Value of the Doctorate in Dentistry, 400.
Thompson, Alton Howard, D.D.S. A Manual of Comparative Dental Anatomy for Dental Students, 472.
Three Thousand Questions on Medical Subjects arranged for Self-Examination, 267.
Three Years none too long for a Dental Course, 778.
Time Limit in Professional Work, 620.
Tincture of Iodine for the Removal of Deposits, 406.
Tin-Foil in Model and Bite Impressions, Use of, 211.
Tooth-Extraction, Death from Attempt at, 481.
Tonsils, False, 345.
To the Alumni of the Dental Department of the University of Maryland, 555.
"Tothe-Lore," 1.
Transactions of the Iowa State Dental Society at the Thirty-fifth Annual Meeting held at Des Moines, May, 3, 4, 5, and 6, 1898, 268.
Treatment for Hemorrhage, The New, 224.
Treatment of Carbolic Acid Poisoning, 620.
Treatment of Pyorrhœa Alveolaris, 442.
Treatment of Pyorrhœa in Detail, 446.
Treatment of Suppuration by Bicarbonate of Soda, 29.
Trueman, William H., D.D.S. On Dental Organizations: Their Mistakes and Failures, 557.
  On Dental Science in 1480, as expounded by Peter de Largelata, 712.
Truman, James, D.D.S. On Reflexes from Lower Molars, 641.
  Remarks, 447.
Turpentine, 817.
Two Cases of Diseased Antrum, 163.

U.

Union Meeting, Seventh and Eighth District Dental Societies, State of New York, 696.
Unusual Effect of Cocaine, 771.
Use of Tin-Foil in Model and Bite Impressions, 211.

V.

Value of the Doctorate in Dentistry, The, 400.
Vital Tissue, Is Enamel? 621.
Vocal Hinderances, 213.
"Volasen," 833.

W.

Waters, Lithia, 101.
Western Criticism, 327.
What constitutes just Criticism? 264.
White, Harry F., On Pyorrhœa Alveolaris, 441.
Whitney, Dr. J. M. On Necrosis from Lactic Acid Injections, 220.
Willoughby, E. F., M.D. Prophylactic Inoculation, 781.
Wins, The Medico-Chi, 339.
Wisconsin State Dental Society, 489.
Wisdom-Tooth, Death from cutting, 771.
Work of the Foreign Relations Committee, 403.

Y.

Younger, W. J., M.D. On Pyorrhœa Alveolaris, from a Bacteriological Stand-Point, with a Report of some Investigations, and Remarks on the Treatment, 413.

Z.

Zentler, Dr. Arthur. On A Case of Triple Reimplantation, 293.

